# Effect of Personalized Nutrition on Dietary, Physical Activity, and Health Outcomes: A Systematic Review of Randomized Trials

**DOI:** 10.3390/nu14194104

**Published:** 2022-10-02

**Authors:** Sangeetha Shyam, Ke Xin Lee, Angeline Shu Wei Tan, Tien An Khoo, Shivani Harikrishnan, Shehzeen Alnoor Lalani, Amutha Ramadas

**Affiliations:** 1 Centre for Translational Research, IMU Institute for Research and Development (IRDI), International Medical University (IMU), Jalan Jalil Perkasa 19, Bukit Jalil, Kuala Lumpur 57000, Malaysia; 2 Universitat Rovira i Virgili, Departament de Bioquímica i Biotecnologia, Unitat de Nutrició Humana, 43201 Reus, Spain; 3 Pere Virgili Health Research Institute (IISPV), Sant Joan University Hospital in Reus, 43204 Reus, Spain; 4 Consorcio CIBER Fisiopatología de la Obesidad y Nutrición (CIBERObn), Instituto de Salud Carlos III (ISCIII), 28029 Madrid, Spain; 5 Jeffrey Cheah School of Medicine and Health Sciences, Monash University Malaysia, Bandar Sunway 47500, Malaysia; 6 Warwick Medical School, University of Warwick, Coventry CV4 7HL, UK; 7 Dalhousie Medicine DMNS, Dalhousie University, 5849 University Avenue, Halifax, NS B3H 4R2, Canada

**Keywords:** personalized nutrition, nutrigenetics, nutrigenomics, nutrition intervention

## Abstract

Personalized nutrition is an approach that tailors nutrition advice to individuals based on an individual’s genetic information. Despite interest among scholars, the impact of this approach on lifestyle habits and health has not been adequately explored. Hence, a systematic review of randomized trials reporting on the effects of personalized nutrition on dietary, physical activity, and health outcomes was conducted. A systematic search of seven electronic databases and a manual search resulted in identifying nine relevant trials. Cochrane’s Risk of Bias was used to determine the trials’ methodological quality. Although the trials were of moderate to high quality, the findings did not show consistent benefits of personalized nutrition in improving dietary, behavioral, or health outcomes. There was also a lack of evidence from regions other than North America and Europe or among individuals with diseases, affecting the generalizability of the results. Furthermore, the complex relationship between genes, interventions, and outcomes may also have contributed to the scarcity of positive findings. We have suggested several areas for improvement for future trials regarding personalized nutrition.

## 1. Introduction

Unhealthy diets have long been established as contributors to the escalating prevalence of non-communicable diseases, including obesity, diabetes, cardiovascular disease, and cancers in a population. These diseases are often major contributing factors to mortality and morbidity [1,2,3]. Thus, current population and individual-based approaches aim to prevent and manage risks in populations or individuals with the underlying understanding that modifiable lifestyle-related factors, including diet and nutrient intakes, have a significant role in the etiology and progression of diseases [3]. These approaches are governed by the assumption that a universal dietary recommendation would work similarly in all individuals and, therefore, in a population. However, this approach is thought to conveniently ignore the inter-individual variations in dietary response that are increasingly being reported [4].

Consequently, it is unsurprising that despite knowledge and research into the link between disease and diet, most dietary interventions and alterations achieve a relatively modest and limited effect on health outcomes, demonstrating the challenges of universal approaches [2,5,6]. With the advent of technological advances in data capture and analysis, there is an increasing awareness that one size may not fit all. Increasing interest in precision and personalized medicine are paralleled by exploration in personalizing nutrition recommendations for individuals.

Personalized nutrition targets prevent diseases and maintain good health using nutritional recommendations tailored to an individual [7,8,9,10]. It is derived from the concept that inter-individual differences exist in response to nutrition and dietary patterns. These differences are attributed to individual variations in biochemistry, metabolism, genetics, and microbiota [7]. Owing to the underlying similarity and overlaps in applying the above concept, the terms personalized nutrition, precision nutrition, nutrigenetics, nutrigenomics, and stratified nutrition are often used interchangeably. However, nuanced differences exist in their approaches [11]. Precision nutrition provides personalized nutritional recommendations based on interacting factors in a person’s internal and external environment. It is based on genetics, epigenetics, food habits, microbiota, metabolome, physical activity, lifestyle factors, and their interactions [12]. The recommendations oftentimes account for the interactions between nutrients and biological processes in an individual, resulting in differential responses to food-derived nutrients [7]. 

Personalized nutrition is purported to be more beneficial than more generalized approaches based on two main concepts. First, genotypic and phenotypic characteristics differ among individuals and cause differential responses to food and nutrients. This implies that there theoretically exists a specific diet that is most beneficial for a specific individual. The second idea is that an appropriate intervention recommended after analyzing one’s current behavior, preferences, and objectives will motivate and better enable individuals to make decisions and changes to their eating patterns [8]. This, in turn, would mean better compliance with the recommendations. Therefore, hypothetically, personalization in such interventions may be more beneficial in achieving health goals [2]. While personalized nutrition remains a topic of interest among scholars, its adoption into current practice is limited. For widespread adoption of personalized nutrition, there is a need to collate and review current evidence on the impact of personalized nutrition on health outcomes. 

Acknowledging this paucity of evidence on the benefit of personalized nutrition, we conduct a systematic review of published randomized trials on the effect of personalized nutrition on dietary intake, physical activity, and various health outcomes. The extracted data is synthesized qualitatively, noting specific trends in populations, age groups, and settings where personalized nutrition interventions have been compared with conventional nutrition interventions using randomized-controlled trials (RCTs). This review collates details on intervention delivery characteristics, including the intervention provider, intervention duration, and delivery settings, and identifies their potential impact, if any, on intervention outcomes. This review also aims to identify health outcomes that show improvement with personalized nutrition and those that show no additional benefit. Finally, this systematic review will assess the quality of the available evidence and identify gaps in the literature on the effectiveness of personalized nutrition and identify areas for improvement in future trials in this area.

## 2. Materials and Methods

### 2.1. Study Design

We used the updated Preferred Reporting Items for Systematic reviews and Meta-Analyses (PRISMA) 2020 Statement [13] and checklist (Supplementary Table S1) to guide this systematic review. The review protocol has been registered with the International Prospective Register of Systematic Reviews (PROSPERO) (CRD42021282746) and is publicly available via https://www.crd.york.ac.uk/prospero/display_record.php?RecordID=282746 [accessed on 25 October 2021].

### 2.2. Search Strategy

We systematically searched the literature in seven electronic bibliographic databases (OVID Medline, PubMed, CINAHL Plus, Scopus, Embase, Cochrane Central Register of Controlled Trials, and ScienceDirect). The following keywords and Boolean operators were used to build the search strategy (“personali?ed nutrition” OR “individuali?ed nutrition” OR “precision nutrition”) AND (genomic* OR gene* OR genetic* OR phenotype OR genotype OR DNA). 

The search was conducted from a journal’s inception to August 2021 and limited to “human studies” and “randomized-controlled trials” where possible. We imposed no language restrictions. A complete database search strategy is shown in Supplementary Table S2.

### 2.3. Study Selection

The study selection process was managed using the Covidence platform [14]. Records obtained from database searches were uploaded to Covidence, and duplicated records were automatically removed. The title and abstracts were first screened according to eligibility criteria. This was followed by the screening of full texts. Two authors (K.X.L. and S.H.) conducted both screening processes independently, and any arising conflicts were resolved by a third author (A.R.). 

We included all randomized trials that reported the effect of personalized nutrition-based dietary intervention on any nutrition or health outcome. This includes individualized nutrition intervention based on an individual’s genetic information gathered at baseline. There were no restrictions with regard to the demography and phenotype of the participants, length of intervention, or geographical location. However, we excluded studies that only gathered genetic information post-intervention. Studies that did not report dietary, physical activity, or health outcomes were also excluded. Grey literature and non-peer-reviewed publications such as book chapters, online abstracts, and conference proceedings were also excluded. 

Five authors (S.A.L., S.H., T.A.K., S.S., and A.S.W.T.) hand-searched the reference lists of the included studies and past reviews to seek articles not identified in the database searches.

### 2.4. Quality Assessment

A quality assessment in the form of a risk of bias analysis on the finalized articles was carried out using the Risk of Bias Assessment Tool provided by the Cochrane Collaboration [15]. The tool includes information on (i) selection bias (random sequence generation and allocation concealment), (ii) performance bias, (iii) detection bias, (iv) attrition bias, (v) reporting bias, and (vi) other bias.

### 2.5. Data Extraction and Synthesis

Data from eligible papers were extracted using Google sheets. We extracted the study origin, age of participants, sample size, intervention characteristics, outcomes assessed, and, most importantly, findings from the final eligible papers. The data were independently reviewed and verified by two authors (K.X.L. and A.R.). 

No meta-analysis was performed due to the heterogeneous nature of the studies and data. However, the extracted data were qualitatively synthesized, noting specific trends in population, age groups, and settings where personalized nutrition intervention was compared with conventional intervention. We also attempted to identify health outcomes that showed improvement with personalized nutrition and challenges in implementing personalized nutrition-based interventions.

## 3. Results

### 3.1. Study Selection and Characteristics

**Figure 1:**
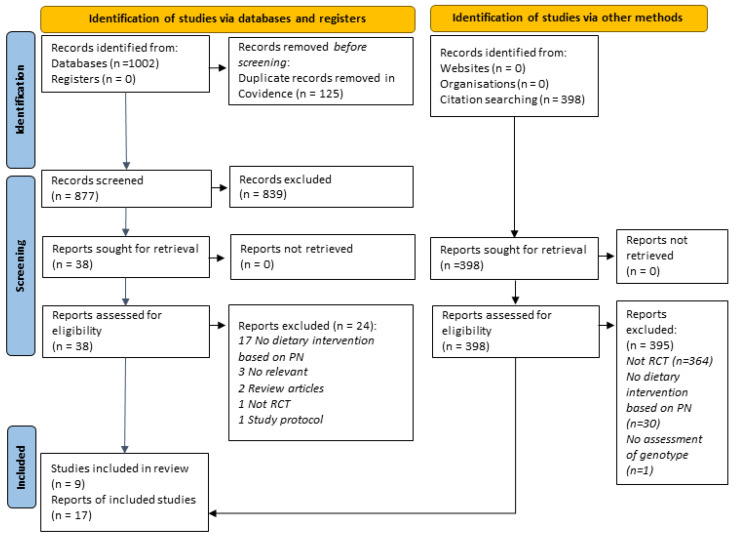
PRISMA 2020 flow chart showing the study selection process.

A total of 1002 records were retrieved from database searches. After the removal of duplicates, 877 titles and abstracts were screened. Subsequently, 42 full texts were sought, and 17 articles (5 unique studies) were finalized after screening according to the review’s eligibility criteria. We found an additional four studies via manual searching. Figure 1 presents the PRISMA flow chart of the study selection. Details of all studies included in the review are provided in Supplementary Table S3. A list of all studies excluded at the full-text screening stage with reasons is available in Supplementary Table S4. 

### 3.2. Study Settings and Population

Nine studies representing a total of 2322 participants [16,17,18,19,20,21,22,23,24,25,26,27,28,29,30,31,32] were included in this systematic review (Table 1). Four of nine studies included in the review were conducted in Canada [18,29,30,32]. Two studies were conducted in the USA [16], while Finland [17] and the Netherlands [31] contributed one trial each. Food4Me was the most extensive study involving 1607 adults between 18 and 79 years, recruited from seven European countries [20,21,22,23,24,25,26,27,28]. Other trials had smaller sample sizes ranging from 51 to 140 participants. Most trials recruited healthy adults [18,20,21,22,23,24,25,26,27,28,29,30,31], while three studies recruited overweight or obese adults [16,19,32]. The participants’ average ages ranged from 22.0 years [29] to 67.7 years [31].

### 3.3. Intervention Characteristics

The majority of the interventions were carried out virtually (N = 5) [17,18,20,21,22,23,24,25,26,27,28,29,30,31], followed by two interventions in primary care [16,31]. The remaining two interventions were carried out in a weight management clinic [19] and a university campus [29]. The duration of the intervention widely ranged from 8 weeks to 12 months. A dietitian or nutritionist delivered the intervention in most trials [16,17,20,21,22,23,24,25,26,27,28,29,30,31].

In general, the intervention participants were provided with a genetic test at baseline and subsequently received personalized advice or knowledge relevant to their genetic test results. For example, in a trial focusing on diabetes-related behaviors [16], participants were provided with a diabetes genetic report consisting of each successfully tested SNP and an overall diabetes genetic risk category. Subsequently, one-on-one genetic counseling sessions were held to explain the genetic test results and contributions of genetic and lifestyle factors to the development of diabetes and to compare the participant’s genetic risk results with their overall diabetes risk. On the other hand, some studies provided feedback to the participants based on pre-determined criteria. The Food4Me trial [20,21,22,23,24,25,26,27,28], in particular, provided feedback based on an algorithm that incorporated genotypic, phenotypic, diet, lifestyle, and anthropometry information. Given the multinational and multicenter nature of the trial, Food4Me utilized an automated online system to achieve this [33]. A more recent trial by Doets and colleagues [31] also took a multiple feedback approach based on a set of nine personalized information categories focusing on diet and physical activity. Interestingly, these researchers used a decision tree based on cut-off values of biological and genetic personalization factors [31].

The intervention can be generally grouped into dietary and lifestyle, disease prevention, and body weight. Grant and colleagues [16] reported the only trial intervening in high-risk participants’ diabetes and body weight through dietary and physical activity changes and genetic counseling. Personalized lifestyle advice was provided to the older adult participants of Doet et al.’s study [31] based on nine underlying decision trees incorporating biological and genetic personalization factors. In another study, a nutrigenic-guided diet was advised for obese veterans to reduce body weight [19]. The Finnish trial among healthy adults focused on lifestyle changes based on the *ApoE* gene [17]. 

The intervention groups are categorized into three groups in the large Food4Me RCT [20,21,22,23,24,25,26,27,28]. The first group was given personalized advice based on current weight, diet, and physical activity. In contrast, the second and third groups were provided with phenotype and genotype information, in addition to the personalized advice. 

The remaining trials provided dietary advice specific to a single or group of nutrients [18,30,31]. A Canadian trial among healthy adults targeted five dietary components, total fat, saturated fat, sugar, omega-3 fatty acids, and sodium [30]. Another trial conducted among healthy Canadian adults provided the participants with personalized genotype-based dietary changes based on caffeine, vitamin C, sugar, and sodium intakes [18]. Roke et al. [29] provide knowledge regarding omega-3 fatty acids and the influence of genetic variation in *FADS*1 for the participants in the intervention group.

### 3.4. Genotype Assessed

Genotype assessment was performed at baseline as the basis for the subsequent individualization of nutrition intervention, with different collection kits utilized and varying combinations of genotypes assessed. 

Thirteen trials assessed lipid-related genotypes, including *FADS1* (endogenous conversion of ALA into EPA and DHA) and NOS3 (risk of elevated triglyceride levels related to omega-3 fat intake), and genotypes associated with lipid metabolism or cholesterol absorption such as *TCF7L2*, *ApoE*, *APOA2*, PPARγ2, and *LIPC* [18,19,20,21,22,25,26,27,28,29,30,31,32]. Genotypes related to cardiovascular health were assessed in 10 trials which include *MTHFR* (folate usage), *ACE* (blood pressure response to sodium intake), and *CYP1A2* (caffeine metabolism) [18,21,22,24,25,26,27,28,30,31]. Obesity and metabolism-related genotypes were also assessed by 10 studies, including *FTO, UCP1, MC4R*, and *ADIPOQ* [19,21,22,23,25,26,27,28,31,32]. Three trials assessed vitamin-related genotypes, namely *GSTT1* and *GSM1* (vitamin C utilization), *VDR Taq1* (vitamin D), and *MMAB* (vitamin B12 metabolism) [18,19,31]. Two studies incorporated genotypes associated with sweet taste perception, *Tas1R2* and *KCTD10,* respectively [18,19]. One study assessed 36 diabetes-related SNPs, which were not specified [16]. *GDF5*, a genotype associated with endurance and resistance training, was assessed in one of the studies [31].

### 3.5. Dietary Outcomes

#### 3.5.1. Diet Quality

Four studies reported the impact of personalized intervention on diet quality [21,22,25,27,30]. Almeida et al. [30] reported significant differences between groups over time for Healthy Eating Index-Canadian (HEI-C) scores, showing more significant improvement in the intervention group who received a personalized nutrition plan that integrated information about their gene test results, health information, personal goals, and dietary intakes. 

However, findings from the Food4Me trial concerning diet quality were largely inconsistent. Livingstone et al. [21] showed MedDiet scores at six months to be greater in individuals who received personalized intervention based on diet, phenotype, and genotype compared with advice based on diet and phenotype alone. Later in 2020, an improvement in HEI score in intervention participants who were carriers of the *MTHFR* risk allele was also observed [27]. On the contrary, two articles noted that including phenotypic and genotypic information does not significantly change HEI in participants receiving personalized nutrition advice [22,25]. 

#### 3.5.2. Dietary Fat

Horne and colleagues [32] found that only the intervention group showed a significant reduction in total dietary fat intake and saturated fatty acid intake at 12 months, while the control group did not significantly change their dietary fat intake long-term. The intervention group had significantly greater adherence to the group-based target for total fat and to targets of <25% kcal from total fat and <10% kcal from saturated fat. Similarly, another Canadian study [30] also observed group differences in the percentage of calories from total fat and saturated fat. 

The Food4Me trial reported no significant differences in dietary saturated fatty acids between *ApoE* risk and non-risk group [20]. However, reductions in the percentage of total fat and saturated fatty acids were observed for those receiving advice based on genotypic data [28]. 

Hietaranta-Luoma and colleagues [17] reported improvement in dietary fat quality by increasing their intake of unsaturated following advice based on the *ApoE* gene as a risk information marker. However, this effect faded after 10 weeks of intervention. Roke et al.’s trial of young adults [29] found that providing participants with *FAS1* genetic information did not differentiate dietary intake of EPA and DHA between the intervention and control groups. However, knowing their *FAS1* status changed perceptions and behaviors related to omega-3 fatty acids. 

#### 3.5.3. Dietary Sugar and Salt

No significant impact on sugar consumption was observed in the reviewed trials [18,24]. Specifically, it was interesting to note that Neilsen and El-Sohemy [18], who intervened with participants with the *Tas1R2* allele associated with increased risk of over-consuming sugars, did not successfully reduce added sugar intake in the intervention compared to controls. 

In contrast, three trials found a significant reduction in salt intake following tailored dietary advice [18,28,30]. A significant reduction in sodium intake was observed in the intervention group who possessed a risk version of the *ACE* gene and were advised to limit their sodium intake, with an increase in the proportion who met the targeted recommendation of 1500 mg/day from 19% at baseline to 34% after 12 months, as opposed to the control group which showed no significant changes [18]. Almeida and colleagues [30] reported significant differences between groups over time for sodium among those who possessed the risk genotype and received tailored dietary advice. Results from the Food4Me trial also found a reduction in salt intake at month 6 for those randomized to receive personalized nutrition advice based on genotype [28].

#### 3.5.4. Other Nutritional Impacts

There is no significant improvement in the folate intake of folate-rich foods in the Food4Me trial [24] from baseline to the sixth month of intervention among those with the *MTHFR* allele. Nielson and El-Sohemy [18] reported no significant changes in vitamin C intake. However, this is likely due to the baseline intake of vitamin C already achieving the recommended intake. The study also did not find any changes in caffeine intake in participants with the *CYP1A2* risk allele associated with an increased risk of myocardial infarction and hypertension when consuming above 200 mg of caffeine/day [18]. 

There was no significant improvement in carotenoids among participants from Germany or *ApoE(rs7412)* genotype carriers in the Food4Me trial [27]. In addition, the trial reported significant improvement in the omega-3 index in participants who successfully changed their physical activity level, reported moderate to vigorous physical activity (MVPA), and have the *ApoE(rs429358)* genotype.

### 3.6. Physical Activity Outcomes

The Finnish study on personalized nutrition based on the *ApoE* genotype reported no significant improvement in leisure-time physical activity between the ε4+, ε4−, and control groups [17]. Assessment of diet quality using different indicators (MedDiet and HEI scores) in the Food4Me trial resulted in the conflicting impact of the intervention on physical activity. Significant differences in moderate and vigorous physical activity among participants with high MedDiet scores compared to participants with low MedDiet scores but not the time spent in sedentary behavior and physical activity level were observed [21]. In contrast, the findings were not replicated in clustering based on HEI scores [27]. In addition, Marsaux et al. [23] showed no significant difference in physical activity in people having or not having the *FTO* risk allele, a gene associated with fat mass and obesity, and provided personalized recommendations.

### 3.7. Health Outcomes

#### 3.7.1. Weight Loss and Anthropometry

The effect of personalized nutrition advice on anthropometry measures varied, and only two studies focused on weight loss or body weight changes [19,21,22,25,26,27]. No significant impact on weight-loss parameters was observed in trials conducted among overweight and obese individuals, which delivered personalized intervention based on a selected set of SNPs related to type 2 diabetes [16] or genes related to components of diet management [19]. Doet and colleagues [31] reported a significant reduction in waist and hip circumferences and body fat percentage but not BMI in older adults. 

The Food4Me trial also reported no significant differences between groups with personalized nutrition or control and between *FTO* gene risk carriers and non-risk carriers of the *FTO* gene, respectively, in terms of body weight, BMI, and waist circumference [25,26]. 

Clustering participants according to dietary intakes resulted in conflicting evidence from the Food4Me trial. According to Livingstone et al. [21], there is a significant but small reduction in waist circumference for participants with high MedDiet scores. At the same time, there are no significant changes in body weight and BMI among both groups with high and low MedDiet scores. The Food4Me trial [22] also reported a better result in participants who met the requirement for oily fish, whole grains, fruits and vegetables, and red meat than those who did not meet the requirement or met only partial requirements. These participants had a healthier diet, lower BMI and waist circumference, and were smoking less. Further assessment of the impact of the Food4Me intervention trial among participants with and without improvement in HEI showed no significant differences in body weight, BMI, and waist circumference [27]. 

#### 3.7.2. Blood Lipids

Frankwich et al. [19] observed no significant difference in the lipid profile (LDL, HDL-cholesterols, and triglycerides) among participants who received genotype-based therapy and standard therapy. The Food4Me trial also did not support significant changes in total cholesterol post-personalized intervention [20,25,27]. 

#### 3.7.3. Quality of Life

Almeida et al. (2019) [30] reported no significant difference in health-related quality of life between the intervention group receiving clustered gene test-based nutrition education and the group providing an integrated practitioner-facilitated method.

### 3.8. Quality Assessment

**Figure 2:**
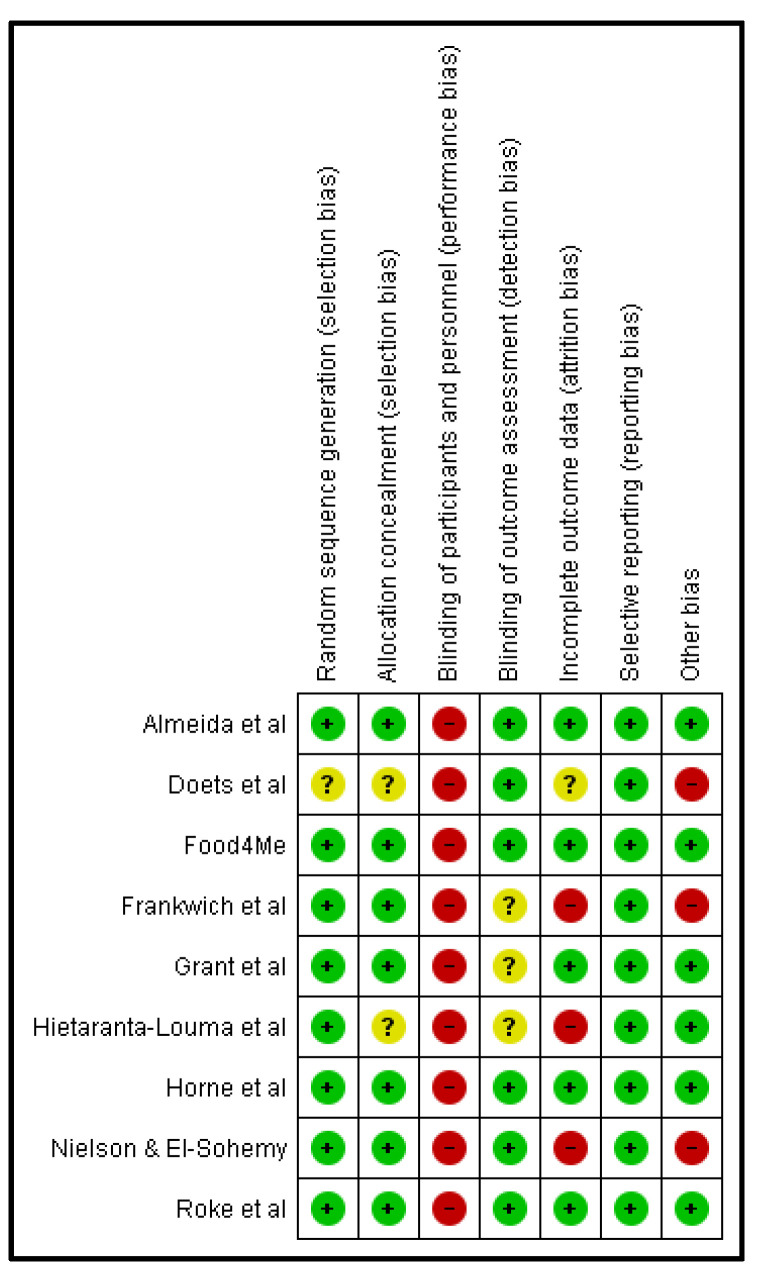
Risk of bias summary of included studies (N = 9) [16,17,18,19,20,29,30,31,32].

Most of the trials were of good quality (Figure 2), though all studies had a high risk for performance bias. This is primarily due to the nature of the intervention, which requires dietary changes. In most studies, participants and personnel could not be blinded to the dietary intervention. Approximately 25% of the trials were at high risk of attrition bias as the intention-to-treat analysis was mostly absent, and several studies did not provide sample size justification.

## 4. Discussion

Given the accruing evidence on inter-individual variations in dietary response [34,35,36], there is increased interest in the additional potential benefit of using personalized approaches to nutrition management. Thus, we systematically reviewed the effect of personalized nutrition on various health outcomes as documented in randomized controlled trials. Most interventions were delivered by healthcare professionals, primarily dietitians, or nutritionists. Only one of the included studies in this review involved a certified genetic counselor as an intervention provider [16]. Five of these interventions [17,18,20,21,22,23,24,25,26,27,28,30,31] were delivered online, whereas the other interventions were delivered face to face in primary care [16,32], weight loss clinics [19], or institutional [29] settings. Most of the studies were funded by national funding agencies, with only two of these studies reporting funding in part from the industry [19,32].

Among the findings, the behavioral effect of personalized nutrition in improving several aspects of dietary intake or physical activity behaviors were either inconsistent or statistically insignificant. The Food4me trial showed that personalized nutrition recommendations improved the omega -3 index in those who performed moderate-to-vigorous physical activity but not in those who were sedentary [27]. The potential biological mechanisms that could explain these discrepancies are not apparent. On the contrary, all three trials that evaluated the effect of personalized nutrition interventions in reducing salt intake showed benefits over the control intervention [18,28,30]. Thus, the most consistent evidence for personalized nutrition in terms of improving dietary intake exists for reducing salt intake and potentially in terms of improving dietary fat quality.

The results of the evaluated personalized nutrition interventions on health parameters, including weight loss, BMI, and waist circumference, were inconsistent. Studies also showed no beneficial effect of personalized nutrition intervention on blood lipids or measures indicating the quality of life. The only beneficial health effect of personalized nutrition was observed in the sole trial [31] that studied the benefit of a personalized nutrition approach in reducing body fat in older adults.

No apparent association of intervention characteristics (the type of intervention delivery (online or face to face), the duration of the intervention, or the study sponsor) with intervention outcome was observed. These findings are not surprising given that health outcomes are not determined merely by gene–diet interactions. It is increasingly understood that what and how much one eats (diet/nutrients), how one eats the diet (dietary pattern, meal combinations, and sequence), when one eats (meal timing, timing restrictions), and other host factors (including gut microbial profile, health or disease condition, age, gender, behavioral and lifestyle factors) are all important in deciding the outcome of a diet for an individual [36,37,38,39]. The results of the current review evaluated specific personalized nutrition recommendations against a control that reflected current standard practice in delivering interventions. Hence, the findings are insufficient to conclude the additional benefits of personalized nutrition interventions and their long-term health impact in comparison to existing standard dietary recommendation practices.

The overall quality of the studies included in this review was moderate to high, with the common limitation being the inability to blind participants and researchers to the intervention allocation. This is a general limitation in nutrition trials and is not specific to those in personalized nutrition. Nevertheless, the findings from this review need to be interpreted cautiously due to several observations. First, the standard of reporting currently among literature reporting on personalized nutrition needs much improvement. There is also the need to better document and report personalized nutrition interventions. Most of the excluded studies in this review did not sufficiently describe dietary intervention. There was an omission of documentation on who delivered the intervention [31]. Process fidelity was also poorly captured among all included trials. Without sufficient description, interventions cannot be reliably implemented, replicated, or built upon [40]. Adopting the template for intervention description and replication (TIDieR) suggested by the Equator network will enable better reporting of interventions in terms of their completeness, specifically for non-drug-related interventions such as personalized nutrition management.

Second, it should be noted that most of the studies were conducted in North America and Europe and had small to moderate sample sizes. While there was a good spread of evidence across categories of the adult life span, intervention in children and adolescents is lacking. Additionally, most of the participants included in these studies were healthy adults. Therefore, further evidence is required to determine the usefulness of personalized nutrition approaches in the treatment or management of diet-related non-communicable diseases. Thus, the generalization of these findings must be carried out with caution.

Third, the genotypes assessed include gene–diet interactions related to lipid metabolism (*FADS1*, *NOS3*, *TCF7L2*, *ApoE*, *APOA2*, PPARγ2, *LIPC*), vitamin metabolism (*GSTT1*, *GSM1*, *VDR Taq1*, *MMAB*, *MTHFR*), and caffeine metabolism (*CYP1A2*). Evaluated genotypes also included those relating to cardiovascular health (*ACE*), obesity and metabolism-related genotypes (*FTO, UCP1, MC4R,* and *ADIPOQ*), sweet taste perception (Tas1R2 and *KCTD10*), and endurance (*GDF5*). One study focused on clustered gene testing related to diet management, weight response, food tolerances, food taste and preferences, and vitamins, minerals, and essential fats [30]. Thus, while the results from these studies are of academic interest, the long-term effects of the personalized nutrition interventions based on narrow genotyping defined by research interest remain poorly understood, given that interactions between various genotypes have not been characterized.

It is argued that personalized nutrition approaches could be more successful as they are tailored to an individual. It is also likely that compliance with such interventions is higher and facilitates long-term maintenance of positive behavioral changes [2,11]. Interestingly, one-third of the trials included in this study had an increased risk of attrition bias, bringing into question the improved compliance expected with the existing personalized nutrition approaches. Additionally, the longest duration documented in the studies included in this review was one year, with many studies ranging between three and four months. Statistically, significant improvements occur after the first six months of the nutritional intervention [41], but the effects of long-term nutritional studies are more modest. This is because maintaining long-term behaviors in the current food environment is challenging [41]. Thus, while most behavioral interventions of medium duration to improve diet and physical activity are reportedly successful, there is a paucity of evidence for longer-term efficacy, which would determine the significance of its impact [41,42].

Given the above concerns, there is a need for robust, well-described personalized nutrition interventions with a well-justified selection of genotyping and spelt-out objectives, specifically in children and adolescents. Most importantly, there is a need to measure diets accurately in such trials [43]. Without accurately measuring what was consumed before the intervention and having a sensitive measurement of the dietary and nutrient changes that happen over time, it is impossible to attribute causality in any nutrition trial, including those in personalized nutrition. Hence, investment in the development of dietary assessment tools and food composition databases is of utmost importance prior to studying the effects of dietary recommendations. The duration of the intervention should also be justified considering the expected endpoints or outcomes, the timeframe of their expected changes, and the sustainability of a benefit, if any. An essential challenge to conducting further investigation is also the sample size required to appropriately power trials to evaluate the benefits of personalized nutrition over conventionally delivered interventions in lieu of the complexity and heterogeneity of host factors and the large measurement errors while measuring dietary exposure, the multiple possibilities of diet–gene interactions, and other confounders [43]. Furthermore, the cost to impact evaluation of personalized nutrition versus conventional approaches requires further investigation to evaluate the justification of adoption into practice for individualized and population-based approaches.

## 5. Conclusions

In this review, we systematically collated the available evidence from randomized controlled trials on personalized nutrition and its impact on health outcomes. Overall, current evidence did not show consistent benefits of personalized nutrition in improving behavioral or health outcomes over the current standard practice. Evidence also does not suggest improved compliance or quality of life resulting from personalized nutrition interventions. With heterogeneity in the gene–diet interactions evaluated, and interventions tested and the lack of a theoretical framework supporting the development of these interventions, the generalizability of the existing evidence, at this time, remains poor.

## Figures and Tables

**Table 1:** Summary characteristics of the included studies (N = 9).

Study, Country	Study Population; Mean Age (Years)	Intervention Focus	Duration of Intervention	Intervention Setting; Provider	Genotype Assessed
Grant et al. [16] USA	108 overweight adults 57.9 ± 10.6	Diabetes prevention behaviors	12 weeks	Primary care Certified genetic counselor, dietitian	A sum of 36 SNPs ^a^
Hietaranta-Luoma et al. [17] Finland	107 healthy adults 47.0 ± 12.1	Lifestyle	1 year	Online Nutritionist, physician	*ApoE*
Nielson and El-Sohemy [18] Canada	138 healthy adults 26.5 ± 3.0	Dietary intake	3 and 12 months	Online Nutrigenomix Inc.	*CYP1A2, GSTT1 and GSTM1, Tas1R2*, *ACE*
Frankwich et al. [19] USA	51 obese veterans 48.4 ± 2.6 (GT group); 54.6 ± 2.7 (ST group)	Weight loss	8 weeks	Weight management clinic Multidisciplinary team	FIT test ^b^
Food4Me [20,21,22,23,24,25,26,27,28] 7 European countries	1607 adults 39.8 ± 13.1	Dietary and diet quality, physical activity, anthropometry, biomarkers changes	6 months	Online Nutritionists and dietitians	*FTO*, *FADS1, TCF7L2*, *ApoE* Ɛ4 and *MTHFR*
Roke et al. [29] Canada	57 young adult females 22.0 ± 1.5	Dietary intake of omega-3 fatty acids	12 weeks	University campus University research team	*FADS1*
Almeida et al. [30] Canada	55 healthy adults 45.8 ± 5.8	Overall dietary changes	9 weeks	Online Dietitian	Clustered gene testing ^c^
Doets et al. [31] Netherlands	59 older adults 67.7 ± 4.8	Lifestyle	9 weeks	Online N/A	*FTO, TCF7L2, FADS1*, *VDR Taq1*, *ACE* and *GDF5*
Horne et al. [32] Canada	140 overweight adults 56.4 ± 12.1 (GLB group); 53.5 ± 13.6 (GLB + NGx group)	Overall dietary changes	12 months	Primary care Dietitian	*UCP1, FTO, TCF7L2, APOA2,* PPARγ2 and *MC4R*

^a^ summary genetic risk score calculated from 36 successfully genotyped risk alleles previously associated with type 2 diabetes; ^b^ a set of SNPs in genes important for obesity, eating behaviors, and exercise; ^c^ gene tests included 5 evidence-based components (diet management, weight response, food tolerances, food taste and preference, and vitamins, minerals, and essential fats).

## Data Availability

Not applicable.

## Supplementary Materials

The following supporting information can be downloaded at: https://www.mdpi.com/article/10.3390/nu14194104/s1, Table S1: PRISMA 2020 Checklist; Table S2: Database search strategy; Table S3: Studies included in the review (N = 9); Table S4: List of studies excluded at full-text COVIDENCE screening with reasons.
