# Supplementary material for: Effect of Personalized Nutrition on Dietary, Physical Activity, and Health Outcomes: A Systematic Review of Randomized Trials
Source: Nutrients. 2022 Oct 2;14(19):4104. doi: 10.3390/nu14194104 (PMC9570623; doi:10.3390/nu14194104)
Supplement: Supplementary file 1 [file nutrients-14-04104-s001.zip › Table S2.pdf]

Table S2. Database search strategy

PUBMED		
**#**	**Searches**	**Results**
1	(((((“personalized nutrition”[All fields] or (“personalised nutrition” [All Fields])) OR (“individualized nutrition” [All Fields]))) OR (“individualised nutrition”[All Fields])) OR (“precision nutrition” [All Fields])	1,271
2	(((((“genetic”[All Fields]) OR (“gene” [All Fields])) OR (“genomic” [All Fields])) OR (“phenotype” [All Fields])) OR (“DNA” [All Fields])	4,419,839
3	((((((“personalized nutrition” [All Fields]) OR (“personalised nutrition” [All Fields])) O (“individualized nutrition” [All Fields]))) OR (“individualised nutrition” [All Fields]))) OR (“precision nutrition” [All Fields])) AND (((((“genetic”[All Fields]) OR (“gene” [All Fields])) OR (“genomic” [All Fields])) OR (“phenotype” [All Fields])) OR (“DNA” [All Fields]))	530
4	((((((“personalized nutrition” [All Fields]) OR (“personalised nutrition” [All Fields])) O (“individualized nutrition” [All Fields]))) OR (“individualised nutrition” [All Fields]))) OR (“precision nutrition” [All Fields])) AND (((((“genetic”[All Fields]) OR (“gene” [All Fields])) OR (“genomic” [All Fields])) OR (“phenotype” [All Fields])) OR (“DNA” [All Fields])) Filters: Randomized Controlled Trial, Humans	24

OVID MEDLINE		
**#**	**Searches**	**Results**
1	personalized nutrition.mp.	332
2	personalise nutrition.mp.	92
3	individualised nutrition.mp.	15
4	individualized nutrition.mp.	103
5	precision nutrition.mp.	135
6	1 or 2 or 3 or 4 or 5	638
7	genetics/mp. or Genetics/	3693430
8	gene.mp. or Genes/	2228344
9	genomics.mp. or Genomics/	80406
10	genotype/ or genotype.mp.	3011008
11	phenotype.mp. or Phenotype/	475983
12	DNA.mp. or DNA/	1629378
13	7 or 8 or 9 or 10 or 11 or 12	4702050
14	6 and 13	268
15	limit 14 to (humans and randomized controlled trial)	23

EMBASE		
#	**Searches**	**Results**
1	personalized nutrition.mp.	771
2	personalise nutrition.mp.	209
3	individualised nutrition.mp.	38
4	individualized nutrition.mp.	192
5	precision nutrition.mp.	237
6	1 or 2 or 3 or 4 or 5	1327
7	genetics/mp. or Genetics/	2769194
8	gene.mp. or Genes/	3843305
9	genomics.mp. or Genomics/	447242
10	genotype/ or genotype.mp.	500821
11	phenotype.mp. or Phenotype/	790143
12	DNA.mp. or DNA/	2134453
13	7 or 8 or 9 or 10 or 11 or 12	6192532
14	6 and 13	617
15	limit 14 to (humans and randomized controlled trial)	52

COCHRANE		
#	**Searches**	**Results**
1	personalized nutrition.mp.	117
2	personalise nutrition.mp.	42
3	individualised nutrition.mp.	15
4	individualized nutrition.mp.	54
5	precision nutrition.mp.	38
6	1 or 2 or 3 or 4 or 5	255
7	genetics/mp. or Genetics/	26691
8	gene.mp. or Genes/	44596
9	genomics.mp. or Genomics/	3345
10	genotype/ or genotype.mp.	14740
11	phenotype.mp. or Phenotype/	7828
12	DNA.mp. or DNA/	20734
13	7 or 8 or 9 or 10 or 11 or 12	84047
14	6 and 13	75
15	limit 14 to (humans and randomized controlled trial)	18

SCIENCEDIRECT	
**Search**	**Results**
("personali?ed nutrition" OR "individuali?ed nutrition" OR "precision nutrition") AND (genomic OR gene OR genetic OR genotype OR phenotype OR DNA)	131

CINAHL+			
#	Searches	Search options	Results
S1	personali?ed nutrition OR TX individuali?ed nutrition OR TX precision nutrition	Search modes – Boolean/Phrase	679
S2	TX gene* OR TX genetic* OR TX genomic* OR TX genotype OR TX phenotype OR TX DNA	Search modes – Boolean/Phrase	1088357
S3	S1 AND S2	Search modes – Boolean/Phrase	333
S3	S1 AND S2	Limiters – Publication Type: Randomized Controlled Trial	23

SCOPUS		
#	Searches	Results
1	(TITLE-ABS-KEY (gene*)) OR (TITLE-ABS-KEY (genetic*)) OR (TITLE-ABS-KEY (genomic*)) OR (TITLE-ABS-KEY (genotype)) OR (TITLE-ABS-KEY (phenotype)) (TITLE-ABS-KEY (DNA))	16167343
2	(TITLE-ABS-KEY (personali?ed AND nutrition)) OR (TITLE-ABS-KEY (individuali?ed AND nutrition)) OR (TITLE-ABS-KEY (precision AND nutrition))	6530
3	((TITLE-ABS-KEY (gene*)) OR (TITLE-ABS-KEY (genetic*)) OR (TITLE-ABS-KEY (genomic*)) OR (TITLE-ABS-KEY (genotype)) OR (TITLE-ABS-KEY (phenotype)) (TITLE-ABS-KEY (DNA)) AND (TITLE-ABS-KEY (personali?ed AND nutrition)) OR (TITLE-ABS-KEY (individuali?ed AND nutrition)) OR (TITLE-ABS-KEY (precision AND nutrition)))	2212
4	((TITLE-ABS-KEY (gene*)) OR (TITLE-ABS-KEY (genetic*)) OR (TITLE-ABS-KEY (genomic*)) OR (TITLE-ABS-KEY (genotype)) OR (TITLE-ABS-KEY (phenotype)) (TITLE-ABS-KEY (DNA)) AND (TITLE-ABS-KEY (personali?ed AND nutrition)) OR (TITLE-ABS-KEY (individuali?ed AND nutrition)) OR (TITLE-ABS-KEY (precision AND nutrition))) AND (EXCLUDE (DOCTYPE, “re”) OR EXCLUDE (DOCTYPE “cp”) OR EXCLUDE (DOCTYPE, “ch”) OR EXCLUDE (DOCTYPE, “ed”) OR EXCLUDE (DOCTYPE, “no”) OR EXCLUDE (DOCTYPE, “sh”) OR EXCLUDE (DOCTYPE, “bk”) OR EXCLUDE (DOCTYPE, “le”) OR EXCLUDE (DOCTYPE, “cr”) AND (LIMIT-TO (EXACTKEYWORD, “Human”)) AND (LIMIT-TO (SRCTYPE, “j”))	732
