# Supplementary material for: Effect of Personalized Nutrition on Dietary, Physical Activity, and Health Outcomes: A Systematic Review of Randomized Trials
Source: Nutrients. 2022 Oct 2;14(19):4104. doi: 10.3390/nu14194104 (PMC9570623; doi:10.3390/nu14194104)
Supplement: Supplementary file 1 [file nutrients-14-04104-s001.zip › Table S4.pdf]

Table S4. List of studies excluded at full-text COVIDENCE screening with reasons

Database search		
#	Citation	Reason
1	Aganzo M, Montojo MT, López de Las Hazas MC, Martínez-Descals A, Ricote-Vila M, Sanz R, González-Peralta I, Martín-Hernández R, de Dios O, Garcés C, Galdón A, Lorenzo Ó, Tomás-Zapico C, Dávalos A, Vázquez C, González N. Customized dietary intervention avoids unintentional weight loss and modulates circulating miRNAs footprint in Huntington's Disease. Mol Nutr Food Res. 2018;62(23):e1800619. doi: 10.1002/mnfr.201800619.	No dietary intervention based on PN was reported
2	Artemyeva NO, Svechnikova EV, Nemchaninova OB, Sklyanova EY. Individualized nutritional approach for atopic dermatitis. Klinicheskaya Dermatologiya i Venerologiya 2020;19(4):528-533.	Not a randomized trial
3	Barrón-Cabrera E, González-Becerra K, Rosales-Chávez G, Mora-Jiménez A, Hernández-Cañaveral I, Martínez-López E. Low-grade chronic inflammation is attenuated by exercise training in obese adults through down-regulation of ASC gene in peripheral blood: a pilot study. Genes Nutr. 2020;15(1):15. doi: 10.1186/s12263-020-00674-0.	No dietary intervention based on PN was reported
4	Calancie L, Keyserling TC, Taillie LS, Robasky K, Patterson C, Ammerman AS, Schisler JC. *TAS2R38* Predisposition to bitter taste associated with differential changes in vegetable intake in response to a community-based dietary intervention. G3 (Bethesda). 2018;8(6):2107-2119. doi: 10.1534/g3.118.300547.	No dietary intervention based on PN was reported
5	Campbell KL, Ash S, Bauer JD. The impact of nutrition intervention on quality of life in pre-dialysis chronic kidney disease patients. Clin Nutr. 2008;27(4):537-44. doi: 10.1016/j.clnu.2008.05.002.	No dietary intervention based on PN was reported
6	Celis-Morales C, Livingstone KM, Marsaux CF, Forster H, O'Donovan CB, Woolhead C, Macready AL, Fallaize R, Navas-Carretero S, San-Cristobal R, Kolossa S, Hartwig K, Tsirigoti L, Lambrinou CP, Moschonis G, Godlewska M, Surwiłło A, Grimaldi K, Bouwman J, Daly EJ, Akujobi V, O'Riordan R, Hoonhout J, Claassen A, Hoeller U, Gundersen TE, Kaland SE, Matthews JN, Manios Y, Traczyk I, Drevon CA, Gibney ER, Brennan L, Walsh MC, Lovegrove JA, Alfredo Martinez J, Saris WH, Daniel H, Gibney M, Mathers JC. Design and baseline characteristics of the Food4Me study: a web-based randomised controlled trial of personalised nutrition in seven European countries. Genes Nutr. 2015;10(1):450. doi: 10.1007/s12263-014-0450-2.	Study protocol
7	Celis-Morales C, Livingstone KM, Petermann-Rocha F, Navas-Carretero S, San-Cristobal R, O'Donovan CB, Moschonis G, Manios Y, Traczyk I, Drevon CA, Daniel H, Marsaux CFM, Saris WHM, Fallaize R, Macready AL, Lovegrove JA, Gibney M, Gibney ER, Walsh M, Brennan L, Martinez JA, Mathers JC; Food4Me Study. Frequent nutritional feedback, personalized advice, and behavioral changes: Findings from the European Food4Me internet-based RCT. Am J Prev Med. 2019;57(2):209-219. doi: 10.1016/j.amepre.2019.03.024.	No relevant outcomes were reported
8	Chamoun E, Carroll NA, Duizer LM, Qi W, Feng Z, Darlington G, Duncan AM, Haines J, Ma DWL; Guelph Family Health Study. The relationship between single nucleotide polymorphisms in taste receptor genes, taste function and dietary intake in preschool-aged children and adults in the Guelph Family Health Study. Nutrients. 2018;10(8):990. doi: 10.3390/nu10080990.	No dietary intervention based on PN was reported
9	Galmés S, Palou A, Serra F. Increased risk of high body fat and altered lipid metabolism associated to suboptimal consumption of vitamin a is modulated by genetic variants rs5888 (*SCARB1*), rs1800629 (*UCP1*) and rs659366 (*UCP2*). Nutrients. 2020;12(9):2588. doi: 10.3390/nu12092588.	No dietary intervention based on PN was reported
10	Gibney ER. Personalised nutrition - phenotypic and genetic variation in response to dietary intervention. Proc Nutr Soc. 2020;79(2):236-245. doi: 10.1017/S0029665119001137.	Review article
11	Gomez-Delgado F, Delgado-Lista J, Lopez-Moreno J, Rangel-Zuñiga OA, Alcala-Diaz JF, Leon-Acuña A, Corina A, Yubero-Serrano E, Torres-Peña JD,	No dietary intervention based on PN was reported

	Camargo A, Garcia-Rios A, Caballero J, Castaño J, Ordovas JM, Lopez-Miranda J, Perez-Martinez P. Telomerase RNA component genetic variants interact with the mediterranean diet modifying the inflammatory status and its relationship with aging: CORDIOPREV Study. J Gerontol A Biol Sci Med Sci. 2018;73(3):327-332. doi: 10.1093/gerona/glw194.	
12	Gomez-Delgado F, Garcia-Rios A, Alcala-Diaz JF, Rangel-Zuñiga O, Delgado-Lista J, Yubero-Serrano EM, Lopez-Moreno J, Tinahones FJ, Ordovas JM, Garaulet M, Lopez-Miranda J, Perez-Martinez P. Chronic consumption of a low-fat diet improves cardiometabolic risk factors according to the CLOCK gene in patients with coronary heart disease. Mol Nutr Food Res. 2015;59(12):2556-64. doi: 10.1002/mnfr.201500375.	No dietary intervention based on PN was reported
13	Heianza Y, Zhou T, Yuhang C, Huang T, Willett WC, Hu FB, Bray GA, Sacks FM, Qi L. Starch digestion-related amylase genetic variants, diet, and changes in adiposity: Analyses in prospective cohort studies and a randomized dietary intervention. Diabetes. 2020;69(9):1917-1926. doi: 10.2337/db19-1257.	Review article
14	Hiel S, Gianfrancesco MA, Rodriguez J, Portheault D, Leyrolle Q, Bindels LB, Gomes da Silveira Cauduro C, Mulders MDGH, Zamariola G, Azzi AS, Kalala G, Pachikian BD, Amadieu C, Neyrinck AM, Loumaye A, Cani PD, Lanthier N, Trefois P, Klein O, Luminet O, Bindelle J, Paquot N, Cnop M, Thissen JP, Delzenne NM. Link between gut microbiota and health outcomes in inulin -treated obese patients: Lessons from the Food4Gut multicenter randomized placebo-controlled trial. Clin Nutr. 2020;39(12):3618-3628. doi: 10.1016/j.clnu.2020.04.005.	No dietary intervention based on PN was reported
15	Kirwan L, Walsh MC, Celis-Morales C, Marsaux CF, Livingstone KM, Navas-Carretero S, Fallaize R, O'Donovan CB, Woolhead C, Forster H, Kolossa S, Daniel H, Moschonis G, Manios Y, Surwillo A, Godlewska M, Traczyk I, Drevon CA, Gibney MJ, Lovegrove JA, Martinez JA, Saris WH, Mathers JC, Gibney ER, Brennan L. Phenotypic factors influencing the variation in response of circulating cholesterol level to personalised dietary advice in the Food4Me study. Br J Nutr. 2016;116(12):2011-2019. doi: 10.1017/S0007114516004256.	No relevant outcomes were reported
16	Marsaux CF, Celis-Morales C, Fallaize R, Macready AL, Kolossa S, Woolhead C, O'Donovan CB, Forster H, Navas-Carretero S, San-Cristobal R, Lambrinou CP, Moschonis G, Surwillo A, Godlewska M, Goris A, Hoonhout J, Drevon CA, Manios Y, Traczyk I, Walsh MC, Gibney ER, Brennan L, Martinez JA, Lovegrove JA, Gibney MJ, Daniel H, Mathers JC, Saris WH. Effects of a web-based personalized intervention on physical activity in European adults: A randomized controlled trial. J Med Internet Res. 2015;17(10):e231. doi: 10.2196/jmir.4660.	No dietary intervention based on PN was reported
17	McMorrow AM, Connaughton RM, Magalhães TR, McGillicuddy FC, Hughes MF, Cheishvili D, Morine MJ, Ennis S, Healy ML, Roche EF, Tremblay RE, Szyf M, Lithander FE, Roche HM. Personalized cardio-metabolic responses to an anti-inflammatory nutrition intervention in obese adolescents: A randomized controlled crossover trial. Mol Nutr Food Res. 2018;62(10):e1701008. doi: 10.1002/mnfr.201701008.	No dietary intervention based on PN was reported
18	Nielsen DE, Shih S, El-Sohemy A. Perceptions of genetic testing for personalized nutrition: a randomized trial of DNA-based dietary advice. J Nutrigenet Nutrigenomics. 2014;7(2):94-104. doi: 10.1159/000365508.	No relevant outcomes were reported
19	Rajendiran E, Lamarche B, She Y, Ramprasath V, Eck P, Brassard D, Gigleux I, Levy E, Tremblay A, Couture P, House JD, Jones PJH, Desmarchelier C. A combination of single nucleotide polymorphisms is associated with the interindividual variability in the blood lipid response to dietary fatty acid consumption in a randomized clinical trial. Am J Clin Nutr. 2021;114(2):564-577. doi: 10.1093/ajcn/nqab064.	No dietary intervention based on PN was reported
20	Ramos-Lopez O, Cuervo M, Goni L, Milagro FI, Riezu-Boj JI, Martinez JA. Modeling of an integrative prototype based on genetic, phenotypic, and environmental information for personalized prescription of energy-restricted	No dietary intervention based on PN was reported

	diets in overweight/obese subjects. Am J Clin Nutr. 2020;111(2):459-470. doi: 10.1093/ajcn/nqz286.	
21	Ramos-Lopez O, Riezu-Boj JI, Milagro FI, Cuervo M, Goni L, Martinez JA. Models integrating genetic and lifestyle interactions on two adiposity phenotypes for personalized prescription of energy-restricted diets with different macronutrient distribution. Front Genet. 2019;10:686. doi: 10.3389/fgene.2019.00686.	No dietary intervention based on PN was reported
22	Rollo ME, Haslam RL, Collins CE. Impact on dietary intake of two levels of technology-assisted personalized nutrition: A randomized trial. Nutrients. 2020;12(11):3334. doi: 10.3390/nu12113334.	No dietary intervention based on PN was reported
23	Sturgeon KM, Foo W, Heroux M, Schmitz K. Change in inflammatory biomarkers and adipose tissue in BRCA1/2þ breast cancer survivors following a yearlong lifestyle modification program. Cancer Prevention Research 2018;11(9):545-550.	No dietary intervention based on PN was reported
24	Sturgeon KM, Foo W, Heroux M, Schmitz K. Change in inflammatory biomarkers and adipose tissue in *BRCA1/2*$^{+}$ breast cancer survivors following a yearlong lifestyle modification program. Cancer Prev Res (Phila). 2018;11(9):545-550. doi: 10.1158/1940-6207.CAPR-18-0098.	No dietary intervention based on PN was reported
